# Genetic Rescue of the Dinaric Lynx Population: Insights for Conservation From Genetic Monitoring and Individual‐Based Modelling

**DOI:** 10.1111/eva.70045

**Published:** 2025-01-10

**Authors:** Elena Pazhenkova, Matej Bartol, Barbara Boljte, Urša Fležar, Andrea Gazzola, Tomislav Gomerčić, Marjeta Konec, Ivan Kos, Miha Krofel, Jakub Kubala, Ladislav Paule, Mihai Pop, Hubert Potočnik, Barbara Promberger, Robin Rigg, Teodora Sin, Magda Sindičić, Vedran Slijepčević, Astrid Vik Stronen, Ira Topličanec, Tomaž Skrbinšek

**Affiliations:** ^1^ University of Ljubljana Ljubljana Slovenia; ^2^ DivjaLabs Ltd. Ljubljana Slovenia; ^3^ Slovenia Forest Service Ljubljana Slovenia; ^4^ Association for the Conservation of Biological Diversity Focșani Romania; ^5^ University of Zagreb Zagreb Croatia; ^6^ Technical University in Zvolen Zvolen Slovakia; ^7^ Foundation Conservation Carpathia Brasov Romania; ^8^ Slovak Wildlife Society Liptovský Hrádok Slovakia; ^9^ Karlovac University of Applied Sciences Karlovac Croatia; ^10^ Aalborg University Aalborg Denmark

**Keywords:** genetic rescue, individual‐based modelling, *Lynx lynx*, population reinforcement

## Abstract

Inbreeding depression poses a severe threat to small populations, leading to the fixation of deleterious mutations and decreased survival probability. While the establishment of natural gene flow between populations is an ideal long‐term solution, its practical implementation is often challenging. Reinforcement of populations by translocating individuals from larger populations is a viable strategy for reducing inbreeding, increasing genetic diversity and potentially saving populations from extinction. The Dinaric population of Eurasian lynx ( *Lynx lynx* ) has faced high inbreeding levels, with effective inbreeding reaching 0.316 in 2019, endangering the population's survival. To counteract this, population reinforcement was implemented between 2019 and 2023, involving the translocation of 12 individuals from the Carpathian Mountains to the Dinaric Mountains of Slovenia and Croatia. We conducted comprehensive genetic monitoring in this area, gathering 588 non‐invasive and tissue samples, which were used for individual identification and estimation of population genetic parameters. We used stochastic modelling to assess the long‐term viability of the Dinaric lynx population post‐translocation and formulate effective conservation strategies. The model predicts that, despite significant improvement of genetic diversity after translocations, inbreeding will return to critical levels within 45 years. Our results highlight the fact that reinforcement is just the first step and that long‐term genetic management is needed to keep the population from sliding back towards extinction. The Dinaric lynx population serves as a compelling example of genetic rescue. The lessons learnt here will be essential for ensuring the viability of the Dinaric lynx in the future and also provide a useful template for conservation of other populations and species facing similar threats.

## 1 Introduction

In the field of conservation biology, inbreeding is recognised as a serious threat to endangered species, particularly those confined to small, isolated groups (Frankham, Ballou, and Briscoe 2002). Often an inadvertent consequence of population fragmentation and/or reduction in population size, it poses a significant risk to the genetic health and long‐term viability of these populations. Every sexually reproducing organism carries a load of deleterious recessive or semi‐recessive alleles. In a large, outbred population, these alleles do not cause much damage as they are rare, and the likelihood of an individual receiving the same deleterious recessive allele from both parents (causing its phenotypic expression) is low (Charlesworth and Charlesworth 1987). It is, however, quite the opposite for inbred individuals, where both maternal and paternal lineages meet in a recent ancestor. In such individuals there is a high probability of the phenotypic expression of such alleles reducing individual fitness, diminishing survival and reproductive success (Allendorf and Luikart 2009). When this is happening at the population level, it leads to inbreeding depression, which can be a major factor in the extinction of a small population (Keller and Waller 2002).

Historically, conservation efforts in reintroduced and other bottlenecked populations focused mainly on demographic aspects such as population size and structure (Jamieson and Lacy 2012). However, over the years, it has become increasingly clear that genetic considerations must be integrated into the broader framework of reintroduction biology to ensure the long‐term success of conservation efforts. This has given rise to the concept of genetic rescue (GR), an approach that prioritises genetic diversity as a fundamental component of population restoration (Tallmon et al. 2004; Bell et al. 2019). The aim of GR is to reduce the risks of inbreeding depression by introducing new genes from closely related populations, thus enhancing the fitness and genetic health of endangered populations. The correlation between genetic parameters, such as heterozygosity and various demographic parameters, has been well‐documented in numerous wild populations, underscoring the pivotal role of genetics in population health and persistence (Agudo et al. 2012; Terrell et al. 2016; Velando, Barros, and Moran 2015). For small and isolated populations suffering from inbreeding, GR has been reported as an extremely useful strategy (Frankham 2015).

The classic case of the reinforcement of the Florida panther ( *Puma concolor coryi* ) population has demonstrated the substantial potential of GR (Pimm and Dollar Jr. 2006; Johnson et al. 2010). Continued monitoring following GR has shown clear benefits, including increased genetic diversity, reduction of morphological and biomedical indicators of inbreeding depression, and a substantial increase in population size. Additionally, survival rates were notably higher among admixed panthers resulting from mating between translocated female Texas pumas ( *Puma concolor stanleyana* ) and male Florida panthers and subsequent generations of their progeny (Hostetler et al. 2010). These promising outcomes have underscored the value of GR as a conservation strategy with the potential to alleviate the genetic load and reduce the deleterious effects of inbreeding.

Genetic divergence of autochthonous Eurasian lynx ( *Lynx lynx* ; hereafter, lynx) subpopulations started during the late Pleistocene climatic changes and continued during their survival in several different refugia during glacial periods (von Arx et al. 2004; Gugolz et al. 2008, Schmidt, Ratkiewicz, and Konopiński 2011, Lucena‐Pérez et al. 2020). Lynx, like other large carnivores, were heavily persecuted by humans and even exterminated from much of their range in the 19th and beginning of the 20th century (Chapron et al. 2014; Lucena‐Pérez et al. 2020). Since the 1970s, there have been several lynx reintroduction and reinforcement projects in central and western Europe, mostly sourcing animals from the Slovak Carpathians (von Arx et al. 2004, Mueller et al. 2020). Human‐induced habitat modification and fragmentation still have a major impact on the isolation of these lynx populations (Müller et al. 2014; Ripari et al. 2022), which show clear genetic differentiation based on several different molecular markers (Hellborg et al. 2002; Schmidt, Ratkiewicz, and Konopiński 2011; Ratkiewicz et al. 2012; Ratkiewicz et al. 2014; Förster et al. 2018; Lucena‐Pérez et al. 2020).

**FIGURE 1:**
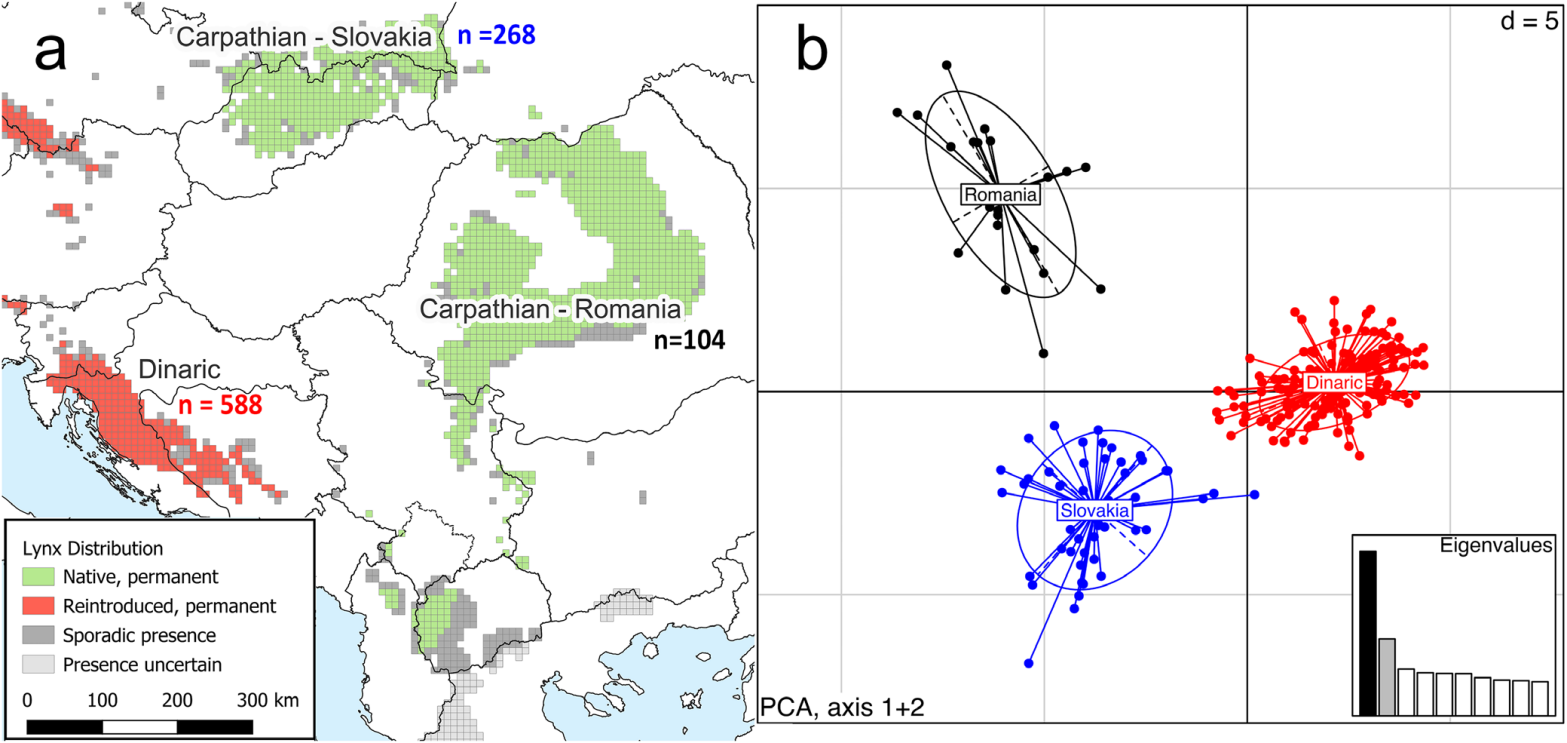
Distribution and genetic structure of studied lynx populations. (a) Ranges of reintroduced and native (source) lynx populations, where *n* represents the number of samples included in the study. (b) Principal component analysis of microsatellite markers. Axes 1 and 2 (with high eigenvalues) show clear structuring both within the Carpathian Mountains as well as the structure caused by reintroduction sampling/genetic drift in the Dinaric Mountains. Axes 1 and 2 represent 12.98% and 6% of the variation, respectively.

The Dinaric population (Figure 1a), which had become extinct at the beginning of the 20th century, was re‐established in south‐eastern Slovenia in 1973 using lynx from the Slovak Carpathians (Čop and Frković 1998; Kos et al. 2004). While the reintroduction initially appeared to be successful, the small number of founders (six individuals, some of them related) led to inbreeding, which resulted in population decline in the 2000s (Kaczensky et al. 2012; Sindičić et al. 2013) and several cases of morphological deformations, some of them lethal and possibly linked to inbreeding, were observed (Krofel et al. 2024). By the 2010s, signs of lynx presence became increasingly scarce, and re‐extinction of the population became a distinct possibility. Genetic studies of the Dinaric lynx after 2010 showed that the population had the lowest genetic diversity of all studied Eurasian lynx populations, although still comparable to other reintroduced populations of this species (Breitenmoser‐Würsten and Obexer‐Ruff 2003; Sindičić et al. 2013; Rueness et al. 2014; Krojerová‐Prokešová et al. 2018), apart from the Harz population in Germany (Mueller et al. 2020). The average inbreeding coefficient exceeded 0.25 (Sindičić et al. 2013), that is an average random breeding event in the population would be equivalent to a direct brother‐sister mating, which could result in considerable inbreeding depression. A recent genome‐wide study of the Eurasian lynx also showed that the Dinaric population has the highest inbreeding by runs of homozygosity (ROH) analysis of all reintroduced populations studied (Mueller et al. 2022).

To prevent a second extinction of the Dinaric population, reinforcement and restoration of lost population connectivity were proposed (Breitenmoser‐Würsten et al. 2007; Zimmermann and Breitenmoser 2006; Kramer‐Schadt et al. 2011; Sindičić et al. 2013, Lucena‐Pérez et al. 2020, Port et al. 2020). The LIFE Lynx project (LIFE16 NAT/SI/000634) therefore aimed to reinforce the remaining population and establish stepping stones. Translocations of 12 individuals to the Dinaric part of Slovenia and Croatia were performed in 2019–2023 to enhance the genetic diversity of the population (Krofel et al. 2024). Without gene flow, natural or assisted, the problem of inbreeding cannot be completely solved in the long‐term, as the effective population size would invariably remain low, with resulting high genetic drift causing inbreeding to continue accumulating. Thus, in order to establish a connection between the Dinaric and Alpine populations, 10 animals were also translocated to the southeastern Alps in Slovenia and Italy in 2021–2023 (Krofel et al. 2024). If this does not lead to gene flow between the Dinaric and Alpine populations, further conservation measures will become necessary. The research described here aims to evaluate the effects of the implemented reinforcement and to identify the most appropriate GR strategies for further genetic management of the Dinaric lynx population. To meet these objectives, we assessed the genetic diversity, inbreeding and effective population size of the Dinaric lynx population until 2019, to track the trends shown in earlier studies (Sindičić et al. 2013), and to evaluate the first effects of the population reinforcement. By comparing these parameters with those obtained using samples from the source population in the Slovak Carpathians, we analysed the impact of genetic drift over time and assessed the effective inbreeding. We then used this baseline genetic assessment to parameterise an individual‐based genetic‐demographic model. Finally, we used this model in simulations to predict the population's long‐term viability under various scenarios, to better understand the potential consequences of different management actions and to devise a long‐term strategy for genetic management of the population.

## 2 Material and Methods

### 2.1 Genetic Sampling

Historical genetic data for the Dinaric lynx population were obtained through sampling of lynx hunting trophies, muscle, blood and non‐invasive samples in Slovenia and Croatia in a previous study (Polanc et al. 2012; Sindičić et al. 2013). We included these published data covering the period from 1979 to 2010 in the analysis (*N* = 90).

In the NW Dinaric Mountains (Slovenia and Croatia), genetic samples have been collected opportunistically since 2010: tissue samples from dead lynx and individuals captured for telemetry, as well as non‐invasive samples. When the LIFE Lynx project began, pre‐reinforcement lynx monitoring was performed in a systematic manner between 2017 and 2019. Most of the non‐invasive samples were collected during snow tracking and visiting known scent‐marking sites. A total of 212 samples were obtained up to 2019. During the period of reinforcement, an additional 376 samples were collected in the Dinaric Mountains between 2019 and 2022. In total, 588 samples were obtained from the area.

We also included samples collected in the Slovak part of the Carpathians (*N* = 268), the source population for the 1973 reintroduction and the Romanian part of the Carpathians (*N* = 104; Figure 1a). Samples from Slovakia and Romania were collected between 2002 and 2019. Tissue and scat samples were stored in 95% non‐denatured ethanol. Urine samples (collected in snow) were stored in DETs buffer, hair samples were stored in sealed bags with desiccant (silica) and saliva samples were collected with forensic swabs with desiccant integrated in the storage tube. Blood samples were taken from animals captured for telemetry using QIAcard (Qiagen) and stored at room temperature.

#### 2.1.1 DNA Extraction

DNA extraction and PCR setup for non‐invasive and historical genetic samples were conducted in a specialised laboratory dedicated to these procedures (Skrbinšek et al. 2010, 2019). DNA extraction from historic samples is described in Polanc et al. (2012), and manual extraction from non‐invasive samples using the Qiagen Stool Kit is described in Skrbinšek et al. (2010, 2019). For all non‐invasive genetic samples collected after 2015, we used the MagMAX DNA Multi‐sample Kit (Thermo Fisher Scientific), following the ‘whole blood’ protocol. The extraction protocol was implemented on a liquid handling robot (Hamilton STARlet). DNA extraction from tissue samples was done using a manual DNA extraction kit (Sigma GenElute Mammalian Genomic DNA Miniprep Kit) following the manufacturer's protocol.

#### 2.1.2 Genotyping

For genotyping, we used a set of 19 microsatellite markers: Fca132, Fca201, Fca247, Fca293, Fca391, Fca424, Fca567, Fca650, Fca723, Fca82, F115, F53, Fca001, Fca132, Fca161, Fca369, Fca559, Fca742, HDZ700 (Menotti‐Raymond et al. 1999, 2005; Williamson et al. 2002). The SRY locus was used to determine the sex of the animal. Microsatellites were amplified in three multiplexes; detailed protocols are described in Polanc et al. (2012). Genotyping of the historic and tissue samples is described in Polanc et al. (2012). For non‐invasive samples, we used a modified multi‐tube approach (Taberlet et al. 1996; Adams and Waits 2007), described in detail in Sindičić et al. (2013). We genotyped samples of 207 different individuals to assess the status of the populations before the start of the reinforcement in 2019: 22 from Romania, 48 from Slovakia and 137 from the Dinaric Mountains. The Dinaric sample set also included the historical samples analysed in Sindičić et al. (2013). Annual lynx monitoring has been undertaken since 2019 (Krofel et al. 2024). In the sampling seasons 2019–2020, 2020–2021 and 2021–2022, 16, 15 and 15 new individuals from the Dinaric Mountains were genotyped, respectively, and added to the dataset to observe changes in the inbreeding estimates during this period. The list of obtained genotypes is available in Table S1.

### 2.2 Estimation of Population Genetic Statistics

#### 2.2.1 Genetic Diversity and Inbreeding

Genetic data were organised in a Microsoft Access database used to keep records of field data connected with genotyping results (Skrbinšek, unpublished). Nuclear DNA diversity was measured as the number of alleles per locus (*A*), observed heterozygosity (*H*_o_) and Nei's unbiased expected heterozygosity (*H*_e_) (Nei 1978), using the R statistical environment (R Development Core Team 2023) with the package ‘adegenet’ (Jombart 2008). We used the same package to explore genetic structure using principal component analysis (PCA). We visually examined eigenvalues in a scree plot to determine the number of interpretable components and plotted the results to explore the patterns of genetic structure.

We applied Wright's hierarchical structuring of inbreeding (Wright 1931) to measure inbreeding in the Dinaric lynx population. As suggested by Keller and Waller (2002), in a randomly breeding population (*F*_is_ = 0), the actual inbreeding that would cause inbreeding depression is equal to *F*_st_, which measures genetic differentiation between the studied population and metapopulation or entire species. In the case of Dinaric lynx, given that the population was reintroduced, the drift component of inbreeding (*F*_st_) directly reflects the inbreeding of the Dinaric population relative to the source population in the Slovak Carpathians. We used the term ‘*effective inbreeding (Fe)*’ (Frankham, Ballou, and Briscoe 2002), where *H*_Din_ denotes the heterozygosity of the Dinaric lynx population and *H*_SK_ refers to the heterozygosity of the source population.

We used a travelling window approach (Sindičić et al. 2013) to explore the erosion of genetic diversity caused by genetic drift in the Dinaric population, illustrating the dynamics of effective inbreeding through time. We used 60 samples as the width of the window.

#### 2.2.2 Effective Population Size

All estimates of effective population size (*Ne*) were done in the program NeEstimator (Do et al. 2014). We used the linkage disequilibrium method (*LD*) to estimate the *Ne* of the Dinaric population and how it changed through time (Waples 2006). The method uses linkage disequilibrium that forms between unlinked loci in a single sample of genotypes. In small populations (*Ne* < approx. 500), this signal becomes strong enough to enable an estimate of *Ne*. To obtain large enough sample sizes, we separated data into groups according to the decade of sampling. For the last decade, a large number of samples were collected, and we were able to separate them into two temporal groups to obtain a better resolution for the period immediately prior to population augmentation. Extensive testing with simulations has indicated that including at least 25 individuals per group should provide enough data for reliable estimates when the actual *Ne* is small (Waples 2006; Waples and Do 2010), and all our sample groups exceeded this threshold. Following recommendations from Waples and Do (2010), we excluded rare alleles with frequencies below 0.02. Waples and Do (2010) conjectured that samples that include several cohorts should correspond to *Ne* in any given generation, which was later supported with simulations by Robinson and Moyer (2013). As our groups of samples randomly included animals of different (unknown) ages collected over a 5–10‐year timespan, each sampling period corresponds to roughly 2–2.5 lynx generations. Consequently, each sample for *LDNe* includes several adjacent cohorts and provides a reasonable estimate of a generational *Ne*.

### 2.3 Forward Time Simulations

We used simuPOP (Peng and Kimmel 2005) to perform individual‐based simulations of population development. We modelled overlapping generations to make simulations closer to the natural system. We simulated the target Dinaric population and two source populations (Romania and Slovakia). The target population had the following parameters: census size, litter size, number of lethal equivalents, kitten survival rate, carrying capacity, mortality and allelic frequencies. Source populations were modelled as hypothetical populations of adult individuals with no mortality, no mating and infinite population size, but with the genotypes sampled from the actual observed allelic frequencies estimated through genetic monitoring.

The attributes assigned to each simulated individual were its age, sex, genotype, identification number (id) and the ids of both parents. At the initial point of simulation, the individual genotypes were randomly sampled from the empirical allelic frequencies estimated for each population (Table S2), and ages were randomly assigned under a negative binomial distribution. Later, new individuals inherited genotypes from parents under Mendelian laws and started from the age of 0. Translocations from the source population to the target population were conducted with a user‐controlled frequency and number of males and females.

Recent estimates showed the population size in the NW Dinarics (i.e. excluding part of Croatia and Bosnia & Herzegovina) as 156 (123–198) with an average density of 1.27 (1.00–1.61) lynx/100 km^2^ (Krofel et al. 2024). The carrying capacity was set as 240 adult animals (Skrbinšek and Krofel 2008). The mating scheme permitted reproduction of individuals older than 2 years, and males could have multiple matings within a single mating season, while females were able to mate only once per season, resulting in a litter size of 2.1 ± 0.9 kittens (Kaczensky 1991). Baseline juvenile mortality was simulated as 0.5, as estimated for the Swiss Jura population (Kaczensky 1991), and applied directly after mating. We distinguished between natural mortality estimated as 0.13 (Andrén et al. 2006) and the probability of survival connected with inbreeding load. Expected survival probability, defined in Nietlisbach et al. (2018; equation 4), was calculated for each individual, where *F* was given as identity by descent (IBD) and *B* as the number of haploid lethal equivalents. The intercept *A* from the original equation (Nietlisbach et al. 2018) corresponds to mortality not related to inbreeding, and it was excluded from the formula since the baseline mortality was applied in the simulations through a separate function. Calculated expected survival probabilities were used as a success probability to create survival events (0—dead, 1—alive), sampled from the Bernoulli distribution.

The simulation was run for 50 generations, where a generation corresponded to the mating season, and averaged over 50 replications. After each generation, we calculated expected and observed heterozygosity, *Ne* and inbreeding coefficient both as identity by descent (IBD) and effective inbreeding (*F*e). At the start of the simulation, individuals already had a high level of inbreeding, but it is hard to estimate the baseline IBD coefficient from the empirical data because kinship would need to be known for almost the entire population. To calculate the baseline IBD, we began the simulations from the reintroduction of six animals from Slovakia, as was done in the original 1973 reintroduction. For this simulation, we performed 500 iterations over 45 years. When we achieved inbreeding coefficient and heterozygosity close to the values observed in the empirical data, we created a distribution of IBD coefficients, from which we sampled IBDs for further simulations. We took into account that the founder animals included a mother and son and assumed that another two animals were siblings, as reported by Koubek and Červený (1997).

We used the Random Forest (RF) algorithm, which was successfully applied to evaluate the predictive power of individual‐based simulations (Edali and Yücel 2019). Due to the continuous nature of the parameters, we used RF regression implemented in python scikit‐learn 1.3.0 (Pedregosa et al. 2011). We fitted the RF metamodel with input model parameters (‘natural mortality’, ‘number of lethal equivalents’, ‘kittens survival rate’, ‘litter size’ and ‘census population size’) as independent variables and the simulation outputs (IBD, *N*e and *H*exp) as dependent variables. The dataset was split into training and validation sets. To validate the performance of the Random Forest model, we assessed the model's predictive accuracy on a validation set using Mean Squared Error (MSE), a metric to quantify the fit between predicted and observed values of population statistics. We estimated feature importance using the mean decrease of impurity (MDI) method, which quantifies the contribution of each feature to the reduction in impurity (Gini impurity) during the construction of individual decision trees within the RF ensemble.

#### 2.3.1 Simulated Reinforcement Scenarios

We selected the year 2019 as the starting point for the simulations, corresponding to the start of the translocations. First, we simulated population development without any conservation actions. Then we tested the effects of the population reinforcement done between 2019 and 2023 (completed population reinforcement), followed by long‐term management scenarios of different translocation frequencies (each 3, 5, 10, 15, 20, 25 years) with animals from Romania or Slovakia.

The further simulated translocations started one lynx generation (5 years) after the end of the completed population reinforcement and ended after 50 years. After that, we continued the simulations for another 40 years to explore the long‐term effect of the genetic rescue and the effects of the interruption of a reinforcement programme. For each translocation frequency, we calculated the minimum number of animals that would need to be integrated into the population to keep it below the inbreeding threshold of 0.15 in the periods between translocations. We fitted the RF metamodel using the translocation frequency, source population and minimum number of animals as independent variables, and IBD, *Ne* and expected heterozygosity as dependent variables to estimate the feature importance for the model as described in the previous section.

## 3 Results

### 3.1 Assessment of the Pre‐Reinforcement Population Genetic Status and Effect of Translocations

#### 3.1.1 Genetic Diversity, Effective Population Size and Effective Inbreeding

A PCA of the microsatellite markers reveals population‐specific clusters on two PCA axes for all three study areas (Figure 1b). It clearly separates individuals from Slovakia and Romania, revealing genetic differentiation caused by spatial isolation and/or distance. There is also a clear differentiation between Dinaric and Slovak lynx, caused by the reintroduction bottleneck and genetic drift in both the source and reintroduced populations, which have been completely isolated since the 1973 reintroduction.

Slovak and Romanian lynx both display considerably higher heterozygosity and allelic diversity compared to the Dinaric population (Table 1). Moreover, the Dinaric population shows a consistent decrease in genetic diversity over time, as illustrated by changes in heterozygosity (Table 1).

**TABLE 1:** Genetic diversity indices for the study areas before the recent lynx reinforcement started (2019) and changes in diversity indices in the Dinaric population through time.

Study area	*N*	*H*_e_ ± SE	*H*_o_ ± SE	*A* ± SE
Slovakia	48	0.583 ± 0.031	0.585 ± 0.034	4.21 ± 0.181
Romania	22	0.545 ± 0.039	0.512 ± 0.04	3.58 ± 0.257
Dinaric	138	0.457 ± 0.036	0.429 ± 0.036	3.21 ± 0.282

Dinaric Mountains, by time periods						
Time period	*N*	*H*_e_ ± SE	*H*_o_ ± SE	*A* ± SE	*Ne* (95%CI)	*Fe* (95% CI)
1979–1990	31	0.481 ± 0.036	0.439 ± 0.036	2.79 ± 0.164	20.3 (12.9–35.5)	0.176 (0–0.36)
1991–2000	26	0.471 ± 0.036	0.477 ± 0.042	2.95 ± 0.209	35.9 (18.7–123.2)	0.192 (0–0.38)
2001–2010	28	0.432 ± 0.04	0.431 ± 0.046	2.74 ± 0.2	11.4 (7.6–17.5)	0.260 (0.02–0.45)
2011–2016	22	0.397 ± 0.038	0.39 ± 0.039	2.58 ± 0.159	13.4 (10.2–17.5)	0.321 (0.1–0.5)
2017–2019	32	0.399 ± 0.039	0.407 ± 0.042	2.63 ± 0.175	13.4 (10.2–17.5)	0.316 (0.1–0.5)

Abbreviations: *A*, allelic diversity; CI, confidence interval; *Fe*, effective inbreeding; *H*_e_, expected heterozygosity; *H*_o_, observed heterozygosity; *N*, number of genotyped individuals; *N*e, effective population size; SE, standard error.

We estimated the linkage disequilibrium *Ne* for the period from 1979 to 2019. Our estimates suggest a dynamic change in *N*e over time. The initial period following the 1973 reintroduction was accompanied by a rapid population expansion, leading to a higher *N*e. This observation aligns with expectations, as growing populations tend to lose less genetic diversity than stable or declining populations, resulting in a higher *N*e (Frankham 2005). However, the *Ne* declined after 2000 and has remained relatively stable at a low level of 13.4 (95% CI: 10.2–17.5) for the last two decades. During the first few generations following the reintroduction in 1973, the small population size necessitated breeding with close relatives, resulting in assortative mating (*F*_is_ > 0). As the population grew, mating became more random; however, inbreeding increased due to the effects of genetic drift. Even in the 1980s, relatively soon after the reintroduction, inbreeding was already comparatively high (*F*_e_ = 0.176). Despite this inbreeding, the population expanded rapidly (Čop and Frković 1998).

**FIGURE 2:**
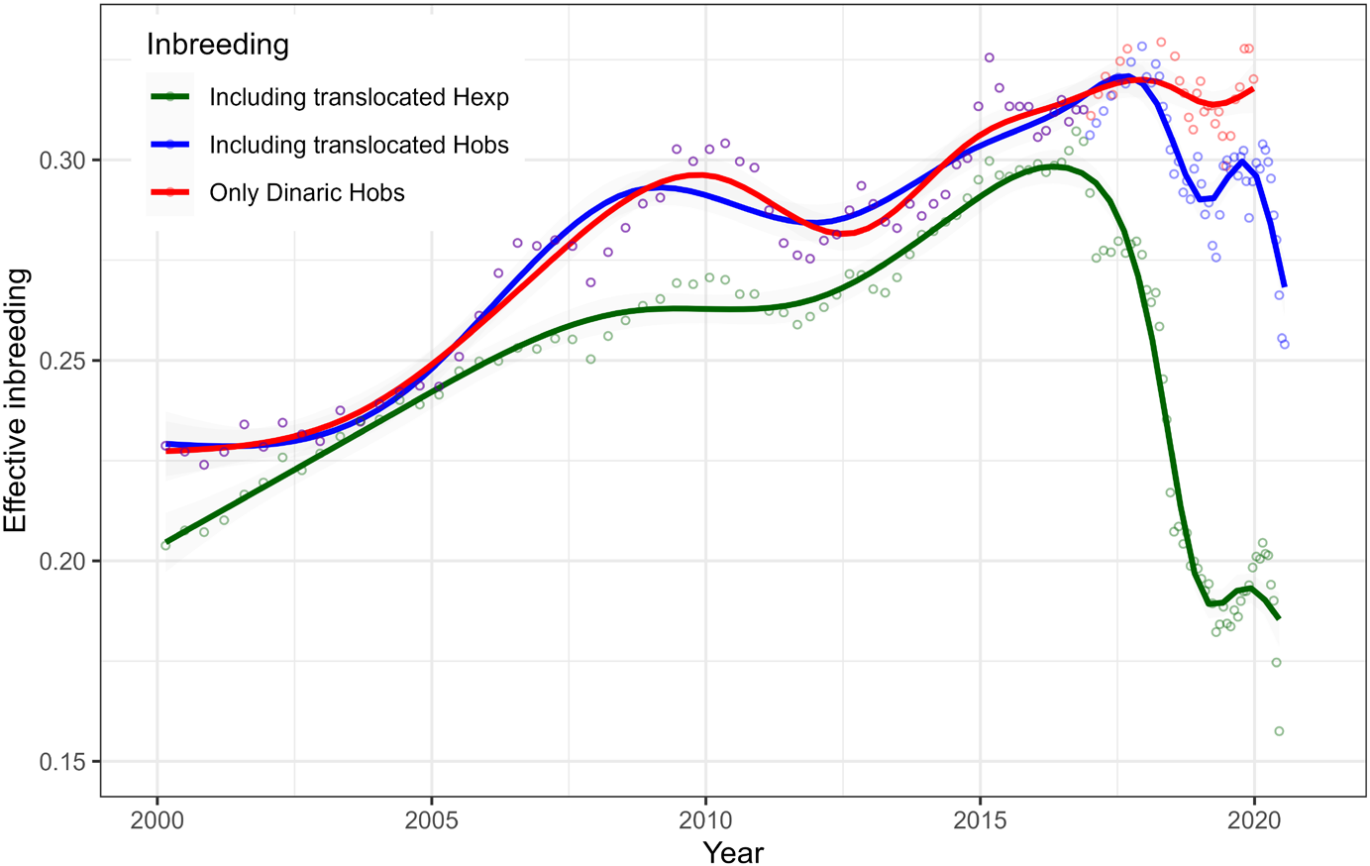
Effective inbreeding (*F*e) of Dinaric lynx relative to the source population in the Slovak Carpathians, calculated using estimated heterozygosity in Slovak lynx and a 60‐sample travelling window. Red – *F*e calculated without translocated lynx and using observed heterozygosity, indicating the situation without translocations; blue – calculated with translocated lynx using observed heterozygosity, indicating the current situation; green – *F*e calculated with translocated lynx using expected heterozygosity, indicating the potential for a rapid decrease in inbreeding if the translocated animals continue to successfully reproduce.

**FIGURE 3:**
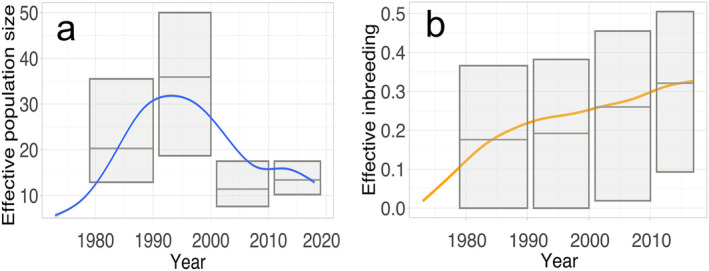
Dynamics of (a) effective population size (*Ne*) and (b) effective inbreeding (*Fe*) of the Dinaric lynx population. Grey lines show empirical estimates and bars represent 95% confidence intervals, coloured lines show modelled values.

However, in the period 2001–2010, inbreeding in the population exceeded that of a brother‐sister mating (*Fe* = 0.260), likely affecting the viability and fecundity of the Dinaric lynx. This trend continued, with inbreeding reaching *Fe* = 0.316 for the last measurement period immediately prior to the reinforcement (2017–2019; Table 1, Figures 2 and 3b).

With more data available, we can confirm that, without translocations, inbreeding in the Dinaric lynx population would have remained consistently high (Figure 2). However, including translocated animals and their offspring in the analysis has led to a substantial reduction in inbreeding. When using observed heterozygosity for calculations, there is an evident drop in *Fe* even though the translocated animals and their offspring do not yet form a large proportion of the population. If the introduced animals and their offspring formed 15% of the total population (simulated through percentage of translocated/offspring animals included in the travelling window sample; green line in years 2019–2020 on Figure 2), the inbreeding estimated from expected heterozygosity would drop to 0.18 and approach 0.15 when translocated animals and their offspring formed around 40% of the population (the right‐most green dots on Figure 2 where recently translocated animals are included). While this value is still high, it falls within the range observed in the 1980s, when the population was still considered viable and expanding. The observed reduction in inbreeding may also be influenced by the Wahlund effect (Wahlund 1928), which occurs when two or more genetically distinct subpopulations are mixed, temporarily increasing heterozygosity. It explains the initial drop in inbreeding when using observed heterozygosity (blue line, Figure 2), even though translocated animals form only a small proportion of the population. However, as these subpopulations merge and interbreed over time, this temporary effect would diminish, and the inbreeding coefficient would stabilise at a lower level, as suggested by the trend seen in *Fe* calculated with expected heterozygosity.

### 3.2 Modelling Population Development

We simulated population development from reintroduction in 1973 until 2018 to evaluate model parameters and to calculate individual IBD coefficients for further simulations. We tested different numbers of lethal equivalents (*B*) ranging from 0.1 to 6, and *B* = 1.1 resulted in *Ne* and *Fe* values close to the empirical estimates.

The dynamics of *Ne* of the Dinaric lynx population show a fluctuating trend over time (Figure 3a). It peaked in the mid‐1990s, then a sharp decline occurred afterward. At the same period, *Fe* started to increase rapidly (Figure 3b). The modelled values align closely with the empirical data.

### 3.3 Population Development in the Absence of Genetic Rescue

**FIGURE 4:**
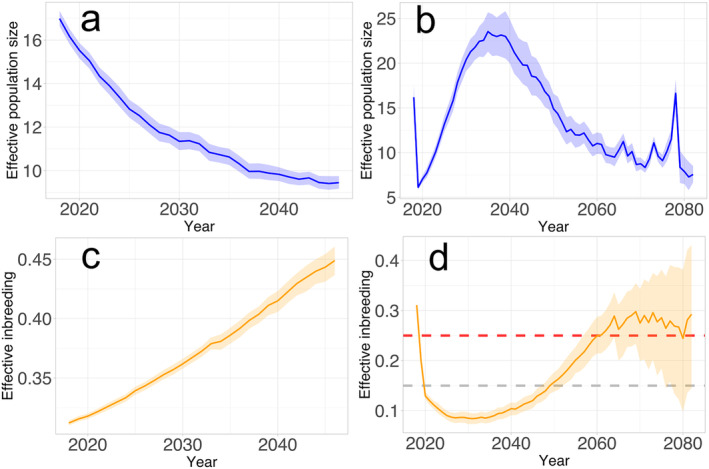
Effective population size and effective inbreeding without (left, panels a,c) and after (right, panels b, d) population reinforcement. The grey dashed line shows the 0.15 threshold, and the red dashed line shows the 0.25 effective inbreeding threshold.

In our simulation modelling of future population development, we also explored the scenario where no translocations were implemented in 2019–2023. Under these conditions, the Dinaric population may be completely extinct on average 28 years after the starting year in the simulations (2018). The model revealed a drastic loss of genetic diversity and a dramatic increase in effective inbreeding, leading to a decrease of *Ne* and eventual population collapse (Figure 4a,c).

### 3.4 Impact of the Implemented Reinforcement

We simulated the population development after translocation for another 60 years and estimated effective inbreeding and *Ne* (Figure 4b,d). The initial part of the graph, demonstrating the changes in *Ne*, showed a sharp, short‐term decrease (Figure 4b), which is an artefact created by the properties of the *LD*‐method for measuring *Ne*. The method tends to have a significant downward bias of *Ne* when two gene pools coexist in the same population, which is observed when individuals from an external population are translocated. The presence of two gene pools elevates linkage disequilibrium (Nei and Li 1973), the main signal for estimating *Ne* with the *LD* method, introducing a bias in the results. However, once the subpopulations had mixed and established a shared gene pool, the *Ne* steadily increased over the subsequent 15 years. Fifty‐three years after the start of the simulations, the *Ne* dropped to a level endangering the population's survival, close to the state prior to the reinforcement efforts.

The translocations significantly delayed the fatal increase in inbreeding and decrease of genetic diversity in the population. For the first 28 years after the reinforcement, the inbreeding level is predicted to remain below the 0.15 threshold. However, after 45 years, the inbreeding level is expected to exceed the 0.25 threshold, equivalent to full‐sib mating, which should prompt an immediate conservation action (Bonn Lynx Expert Group 2021).

### 3.5 Optimisation of Future Genetic Management Strategies

Based on the results of the simulations and the empirical data obtained, the implemented reinforcement had a considerable impact on the genetic parameters of the Dinaric lynx population. As a result, the population is now less likely to succumb to extinction due to genetic erosion. However, to ensure long‐term population viability and prevent a future collapse, it is critical to sustain ongoing conservation efforts (Figure 4b,d).

We compared the effect of integrating animals from Romania and Slovakia through translocations occurring in intervals of 3, 5, 10, 15 and 25 years. For each translocation frequency, we determined the minimum number of animals needed to maintain the population inbreeding below the threshold of 0.15 between translocations (Table 2). To facilitate comparisons between scenarios with different time intervals from the cost and effort perspective, we estimated the total number of lynx that would need to be translocated over 50 years.

**TABLE 2:** Minimum number of animals required per action to be integrated into the Dinaric lynx population to maintain inbreeding levels below the 0.15 threshold between translocations, and the total number of translocated animals per 50 years from two potential source populations at different translocation frequency intervals.

Frequency interval [years]	Slovak source population		Romanian source population	
	*N* per action	*N* per 50 years	*N* per action	*N* per 50 years
3	1	16	1	16
5	2	20	2	20
10	4	20	3	15
15	5	16	4	13
20	5	13	5	13
25	8	16	8	16

In general, translocations from the Romanian Carpathians were more efficient, with 15‐ and 20‐year translocation intervals requiring smaller numbers of individuals. This observation aligns with expectations, considering that the Dinaric and Romanian populations are genetically less similar (Figure 1b). Nevertheless, translocations from the Slovak Carpathia can also be expected to have a substantial positive impact on the genetic diversity of the Dinaric lynx population.

It is important to note that the simulation results provide the minimal number of animals required to prevent inbreeding from reaching critical levels. The model does not consider possible complications during translocations, and our simulations assumed that all of the translocated animals engage in reproduction. A considerable proportion of translocated animals may fail to integrate into the recipient population (Thomas et al. 2023), as was also observed for the 2019–2023 reinforcement of the Dinaric population (Krofel et al. 2024), and any management planning should take this into account.

### 3.6 Metamodel Feature Importance

**FIGURE 5:**
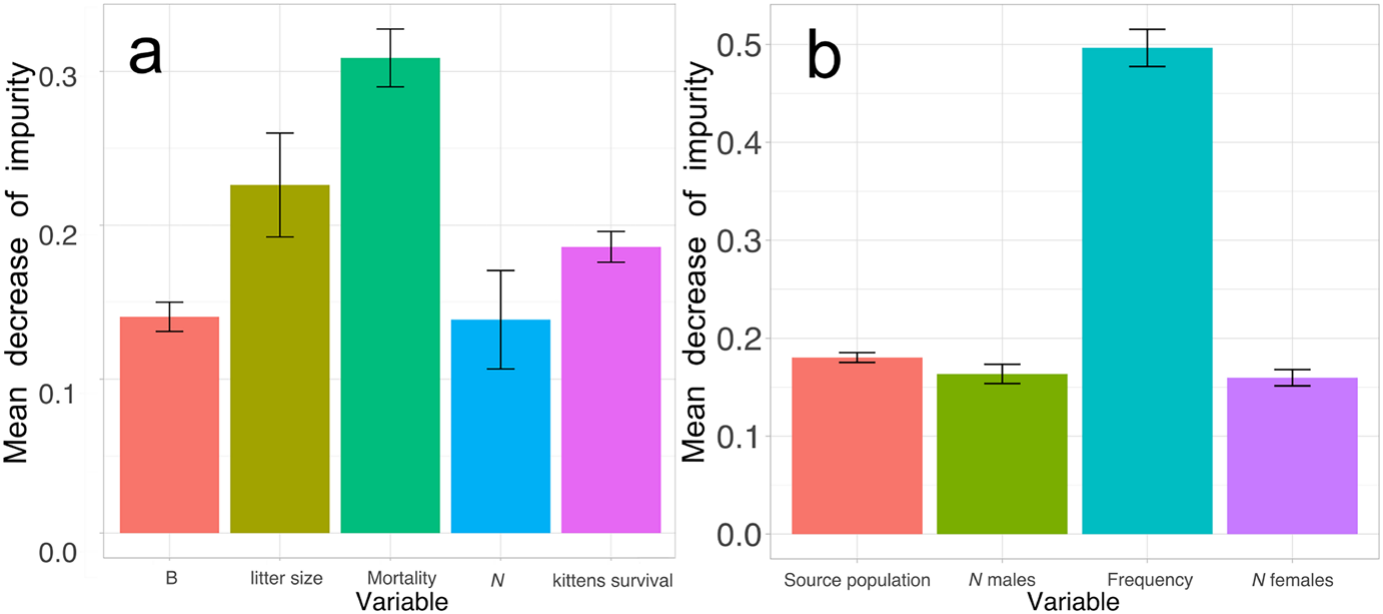
Feature importance for the baseline simulation (a) and translocation scenarios (b). Parameters explored for the baseline simulation were the number of lethal equivalents (*B*), litter size, natural mortality, Dinaric population census size at the start of the simulations (*N*) and the probability of kitten survival. Explored parameters for the translocation scenarios were source population, number of translocated males per translocation, frequency of translocations and the number of translocated females. Averaged MDI values across the 45‐year (a) and 100‐year period (b) of simulation with 95% CIs are shown.

Feature importance scores were obtained after training the RF metamodel. Higher MDI scores indicate a greater contribution of the feature to the model's predictions. Among the model parameters, natural mortality had the strongest influence on the metamodel's predictive power, while census population size had the weakest influence (Figure 5a). A second metamodel investigated the impact of translocation frequency (3, 5, 10, 15, 20, 25 years), the number of translocated individuals (males and females) per translocation event, and the source population. Translocation frequency had the highest MDI score (approximately 0.5), highlighting its substantial influence on the metamodel's predictive accuracy (Figure 5b). This result aligns with the importance of considering the temporal aspect of translocation events in population management. Both regression models exhibited low mean squared error (MSE) (see Tables S3 and S4), indicating close agreement between predictions and actual values, suggesting reasonable model accuracy and precision.

## 4 Discussion

This study demonstrates the adverse effects of inbreeding depression on the Dinaric lynx population and highlights the effectiveness of population reinforcement as a conservation strategy. It provides a window into the population's past and present, but also an insight into its possible future development. As such, the study provides an important foundation for effective long‐term genetic management of the Dinaric lynx population as well as a template for planning long‐term genetic management in similar cases of genetically impoverished populations that need active assistance in order to persist.

Although the Dinaric lynx population expanded rapidly after the 1973 reintroduction, as suggested both by historical data (Čop and Frković 1998) and the increase of *Ne* in that period, it lost a considerable proportion of genetic diversity through the reintroduction bottleneck. As time progressed, so did the genetic erosion, and the population's genetic picture in the years before reinforcement appeared bleak.

The study areas in Romania and Slovakia are spatially relatively far from one another, albeit forming parts of the same Carpathian lynx population (Ratkiewicz et al. 2012; Förster et al. 2018), so it is reasonable to expect a certain level of genetic structuring. Similarly, since the population in the Dinaric Mountains went through a bottleneck, the small number of founders in the 1973 reintroduction and subsequent genetic drift would be expected to create detectable genetic structuring. Similar effects have been observed in other reintroduced lynx populations (Breitenmoser‐Würsten and Obexer‐Ruff 2003; Mueller et al. 2020), as well as in the autochthonous bottlenecked lynx populations in Scandinavia, the Balkans, the Caucasus and Białowieża Primeval Forest in Poland (Bazzicalupo et al. 2021; Hellborg et al. 2002; Ratkiewicz et al. 2014). Nevertheless, when samples from the Dinaric population were included in pan‐European datasets, they clustered together with the source Carpathian population (Förster et al. 2018).

In populations with low *N*e, such as the Dinaric lynx, genetic drift becomes the primary evolutionary force shaping the genetic outlook for the population. Consequently, such populations rapidly lose genetic diversity and become inbred. It leads to a significant reduction of heterozygosity, a trend identified by Sindičić et al. (2013) that has worsened over the past decade. The Iberian lynx (*Lynx paradinus*), which was until recently classified as one of the most endangered felids in the world (Rodríguez and Calzada 2015), demonstrated a similar genetic pattern. By the end of the 20th century, only two isolated populations survived, with *Ne* estimated at around 10 in Doñana and 20 in Sierra Morena. These numbers are comparable to the pre‐reinforcement estimates for the Dinaric lynx (*Ne* = 13.4). Reinforcement efforts, initiated in 2010, have primarily involved releasing captive‐bred individuals, following which Iberian lynx populations are significantly less inbred, with higher genetic diversity than the remnant populations at the end of the 20th century. The Iberian lynx recovery programme has successfully restored relatively low levels of inbreeding and high genetic diversity and the next important step is establishing a connected metapopulation on the Iberian peninsula (Godoy et al. 2024).

Another species of lynx, the bobcat ( *Lynx rufus* ), was reintroduced to Cumberland Island (Georgia, USA), where the population remained isolated for more than 30 years. While observed heterozygosity was relatively high (0.634), estimated *Ne* was comparable to the Dinaric lynx population (*Ne* = 12). Population viability analyses indicated that periodic translocations (e.g. introducing one female every 4 years) would stabilise heterozygosity, underscoring the importance of ongoing genetic management (Miller‐Butterworth et al. 2021).

Reinforcement of Dinaric lynx through the LIFE Lynx project resulted in a significant reduction of inbreeding and an increase in genetic diversity, providing a critical lifeline for the population's survival. In parallel, population decline was reversed, and population abundance increased for > 40% during the project (Krofel et al. 2024). While this demonstrates the efficiency of population reinforcement as a strategy for genetic rescue, the results of the stochastic simulation modelling indicate that these effects take some time to develop as translocated animals become integrated into the population. The improvements achieved are also likely to be relatively short‐lived if they are not followed by gene flow from other populations, either through natural immigration or assisted with additional translocations. Without establishment of gene flow, the simulations predict a decline in population size and an increased risk of extinction for the Dinaric lynx, highlighting the urgency of long‐term proactive genetic management.

The success of the Dinaric lynx population reinforcement can be attributed to several factors. Firstly, the translocated individuals originated from two different population nuclei within a genetically diverse population, ensuring a substantial influx of new genetic variants. Secondly, the translocation process was carefully planned and executed, minimising stress on translocated animals and maximising their chances of successful integration into the Dinaric population (Topličanec et al. 2022). Thirdly, the genetic monitoring conducted during and after the translocations provided valuable insights into the effectiveness of the conservation efforts and facilitated adaptive management strategies (Krofel et al. 2024). Fourth, partnerships with local communities and hunters coupled with transparent communication helped maintain high public and stakeholder support for lynx conservation, preventing the excessive illegal killings (Krofel et al. 2024).

When planning for population reinforcement or long‐term genetic management, selection of the optimal translocation strategy requires careful consideration of several factors:

1. *Translocation frequency*: This should be balanced between maximising the genetic input and minimising the potential disruption to the existing population structure if it is considered to be of evolutionary significance and not just the result of high genetic drift during a recent bottleneck.
2. *Number of translocated individuals*: This should be sufficient to achieve a noticeable impact on population genetic diversity without overwhelming the existing population structure. Translocating a higher number of animals might increase the risk of introducing new maladaptive alleles if the population becomes severely inbred again.
3. *Genetic distance of the source populations*: Selecting genetically distinct source populations can provide a more substantial influx of novel genetic variants, potentially accelerating the recovery of the population. However, it is crucial to consider the potential for outbreeding depression, which can arise when individuals from highly divergent populations interbreed, particularly if they evolved under different ecological conditions.
4. *Source population dynamics*: The impact of translocations on the source population should be carefully evaluated. Excessive removal of individuals from a source population can compromise its genetic diversity and long‐term viability.
5. *Management and logistics*: Longer intervals between translocations increase the likelihood of administrative changes in management plans. While a smaller total number of translocated animals translates to lower overall costs, implementing routine strategies with shorter intervals and smaller groups might be more cost‐effective in the long run. This approach, with optimised parameters, could ultimately lead to lower costs per translocation.

The findings of this study have significant implications for the conservation of endangered populations affected by inbreeding depression. Population reinforcement, when implemented effectively, can serve as a powerful tool to enhance genetic diversity, increase population viability and reduce the risk of extinction. It should be considered a last resort, as it is preferable to maintain natural gene flow between populations whenever possible. However, it is crucial to prevent population extinction while waiting for natural gene flow to be established.

Once it has been deemed essential, population reinforcement should be only the first step, and additional conservation measures should be implemented to support the long‐term viability of the population. For the Dinaric lynx, these include habitat protection and re‐establishing connectivity with neighbouring populations. prey management, mitigation of conflicts with human activities and prevention of human‐caused mortality, such as illegal killing and traffic mortality. The population will also require ongoing monitoring to track its development and detect any increase in inbreeding at an early stage. Finally, as the possibilities of natural gene flow seem limited for now, the population will likely need assisted gene flow through routine translocations from other populations to keep it from sliding towards extinction once again.

The Dinaric lynx population serves as a compelling example of how genetic management can be successfully applied to conserve endangered populations. The combination of population reinforcement, stochastic modelling and genetic monitoring proved to be a valuable approach for assessing the current status of the population, evaluating the effectiveness of conservation interventions and informing future management strategies. This study highlights the importance of proactive genetic management in conservation programmes and provides valuable insights for the conservation of other endangered populations facing similar challenges.

## Conflicts of Interest

The authors declare no conflicts of interest.

## Benefit‐Sharing Statement

We are committed to sharing our research findings with governmental agencies, NGOs, policymakers and local communities involved in lynx conservation. Our publication will be freely accessible to the public through open‐access platforms. We will engage directly with local communities, sharing our findings and facilitating capacity‐building initiatives. Our aim is to maximise the societal impact of our research, promoting collaboration and empowering stakeholders for more effective conservation efforts.

## Supporting information


Table S1.

Table S2.

Table S3.

Table S4.

## Data Availability

The data that supports the findings of this study are available in the supplementary material of this article. Scripts used in this study are available on GitHub: https://github.com/PazhenkovaEA/DinLynx.
